# Supplementary material for: Identification of photoperiod-induced specific miRNAs in the adrenal glands of Sunite sheep (Ovis aries)
Source: Front Vet Sci. 2022 Jul 22;9:888207. doi: 10.3389/fvets.2022.888207 (PMC9354845; doi:10.3389/fvets.2022.888207)
Supplement: Supplementary file 1 [file Data_Sheet_1.ZIP › Supplementary file/miRDeep2 file.zip!/miRDeep2 file/Novel_Structure/LP42A3/Novel_490.pdf]

Provisional ID	:	NC_019463.2_9534
Score total	:	0.4
Score for star read(s)	:	-1.3
Score for read counts	:	-1.3
Score for mfe	:	1.5
Score for randfold	:	1.6
Score for cons. seed	:	
Total read count	:	9
Mature read count	:	9
Loop read count	:	0
Star read count	:	0

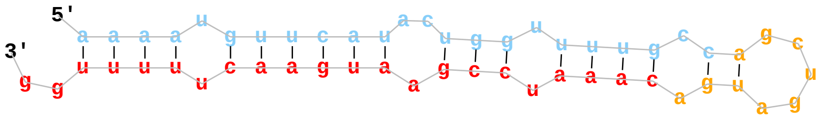

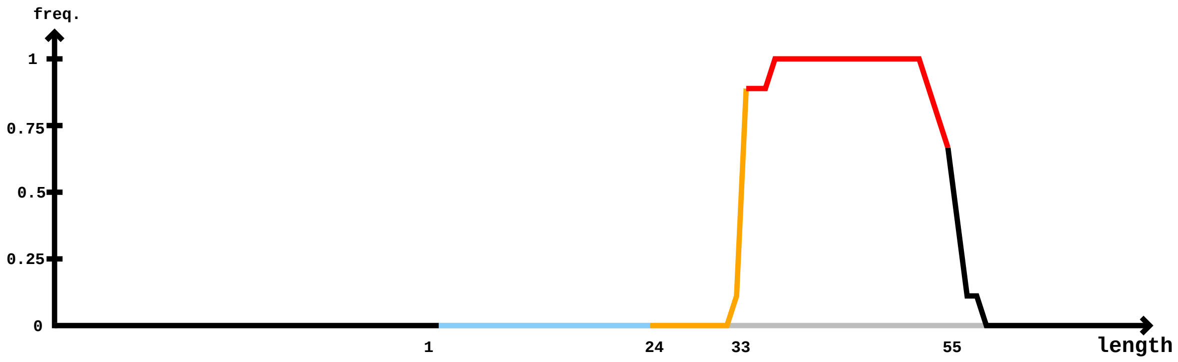


```
                                      Star                   Mature
5'- gcacguacuaggcacucaaaaagaauauuggguaggccaaaauguucauacugguuuugccagcugaugacaaauccgaaugaacuuuuuggccaaccagauauuuguggcg  -3'  exp
    ..........(.(((.......(((((((.(((.((((((((.((((((..(((.((((.((.....)).)))).))).)))))).)))))))).))).)))))))))).).   reads  mm  sample
    .....................................................................aAaaauccgaaugaacuuuuu......................   1      1   seq
    ......................................................................Aaaauccgaaugaacuuuu.......................   1      1   seq
    ......................................................................Aaaauccgaaugaacuuuuug.....................   1      1   seq
    ......................................................................caaauccgaaugaacuuuuugg....................   1      0   seq
    ......................................................................Aaaauccgaaugaacuuuuugg....................   4      1   seq
    .........................................................................auccgaaugaacuuuuuggUca.................   1      1   seq
```
